# A practical solution for preserving single cells for RNA sequencing

**DOI:** 10.1038/s41598-018-20372-7

**Published:** 2018-02-01

**Authors:** Moustafa Attar, Eshita Sharma, Shuqiang Li, Claire Bryer, Laura Cubitt, John Broxholme, Helen Lockstone, James Kinchen, Alison Simmons, Paolo Piazza, David Buck, Kenneth J. Livak, Rory Bowden

**Affiliations:** 1 Wellcome Centre for Human Genetics, University of Oxford, Roosevelt Drive, Headington, Oxford, OX3 7BN UK; 2 Broad Institute of MIT and Harvard, 415 Main St, Cambridge, MA 02142 USA; 3 MRC Human Immunology Unit, Weatherall Institute of Molecular Medicine, University of Oxford, John Radcliffe Hospital, Oxford, OX3 9DS UK; 4 Translational Gastroenterology Unit, Nuffield Department of Clinical Medicine, University of Oxford, John Radcliffe Hospital, Oxford, OX3 9DU UK; 5 Fluidigm Corporation, 7000 Shoreline Ct, South San Francisco, CA 94080 USA

## Abstract

The design and implementation of single-cell experiments is often limited by their requirement for fresh starting material. We have adapted a method for histological tissue fixation using dithio-bis(succinimidyl propionate) (DSP), or Lomant’s Reagent, to stabilise cell samples for single-cell transcriptomic applications. DSP is a reversible cross-linker of free amine groups that has previously been shown to preserve tissue integrity for histology while maintaining RNA integrity and yield in bulk RNA extractions. Although RNA-seq data from DSP-fixed single cells appears to be prone to characteristic artefacts, such as slightly reduced yield of cDNA and a detectable 3′ bias in comparison with fresh cells, cell preservation using DSP does not appear to substantially reduce RNA complexity at the gene level. In addition, there is evidence that instantaneous fixation of cells can reduce inter-cell technical variability. The ability of DSP-fixed cells to retain commonly used dyes, such as propidium iodide, enables the tracking of experimental sub-populations and the recording of cell viability at the point of fixation. Preserving cells using DSP will remove several barriers in the staging of single-cell experiments, including the transport of samples and the scheduling of shared equipment for downstream single-cell isolation and processing.

## Introduction

Single-cell techniques are revolutionising biology by improving the resolution of experiments from the tissue to the cellular level. In particular, the combination of rapid advances in cell isolation technologies, straightforward single-cell RNA sequencing (scRNA-seq) methods, and cheaper high-throughput sequencing, are enabling the capture of detailed information from an ever-larger number of individual cells. The Fluidigm C1™ nanofluidics system is a widely used platform for the isolation and processing of single cells for an increasing range of genomics applications. The C1 platform benefits from ease-of-use, the ability to image cells after capture, and favourable inter-cell consistency, which reportedly is due to its small reaction volumes^1^. A significant unsolved technical issue in single-cell isolation on the C1 (and other platforms) is the maintenance of cell and analyte integrity during preparation of the sample for single-cell isolation. This process can take up to several hours during which the cells are removed from their normal environment. After isolation, molecules such as RNA are typically stabilised by cell lysis in appropriate conditions, so the vulnerable period is the time from initial sample collection to cell lysis. This is a problem that is not unique to single cell studies. Many *in vitro* biochemical analyses suffer from the unresolved concern that manipulation of the sample may be altering the so-called “natural” state of the cells^2^. The ability to “freeze” cell processes as early as possible in the experimental protocol will increase researchers’ confidence that observations represent biological rather than technical effects.

Another factor limiting the feasibility of single-cell studies may be availability of the equipment needed at the time when the cells become available for isolation. This can be because of scheduling conflicts for a limited number of instruments, geographical distance from sample collection to processing location, or clinical procedures that do not fit within normal working hours. The problem of instrument availability is particularly acute for the C1 system. Without multiple C1 instruments, many complex experiments involving replicates, multiple time points, or multiple treatment regimens are impossible because of the difficulty of storing cells, intact, for later isolation and analysis. Treatments enabling the storage of cells in bulk for several days without degradation of RNA, while maintaining the ability to assess viability at the point of initial sample collection, would make it possible to conduct multi-sample experiments on the C1 and other platforms. Researchers have described methods for preservation of cells for single-cell RNAseq by - formaldehyde cross-linking^3^, cryopreservation^4^, and methanol fixation^5^ - with varying combinations for different cell-types, cell-isolation platforms, and cDNA synthesis protocols. These highlight the requirement for sample preservation methods for broadening the scope of single-cell experiments.

Here, we describe the adaptation and testing of a cell-permeable, reversible cross-linking fixative, dithio-bis(succinimidyl propionate) (DSP; Lomant’s reagent), as a cell preservative for single-cell transcriptomic analysis. DSP has been described previously as a reversible fixative for tissue samples preserving their integrity for immunostaining, laser microdissection, and RNA expression profiling with microarrays^6^. We have used DSP for preservation of K562 cells, stained with varying combinations of common dyes in three independent experiments, for subsequent microfluidic isolation and imaging followed by RNAseq library preparation and sequencing. We further assess RNAseq from fixed cells and compare the transcriptome complexity and expression profiles with fresh cells.

## Methods

### Fixation protocol

*Follow these steps to prepare and use DSP for single-cell fixation*.

1. Prepare a 50× stock solution of DSP (50 mg/ml) in 100% anhydrous DMSO.
2. Dispense the stock into 100 μl aliquots and store at −80 °C.
3. Dilute the 50× DSP stock solution to its working concentration (1 mg/ml) with PBS immediately before use, as follows:
  - In a 15 ml Falcon tube, add 490 μl PBS to 10 μl DSP stock dropwise using a 200 μl pipette while vortexing.
  - Check to ensure minimal precipitation; in case of substantial precipitation, start the dilution again with a new DSP stock aliquot.
4. Filter the 1 × DSP using a 30 μm filter (Miltenyi, Pre-Separation Filters; 30 μm).
5. Place 1 × DSP on ice.
6. Dispense 200,000 cells into a 1.5-ml Eppendorf tube.
7. Pellet cells by centrifuging for 5 min at 200 × g and remove supernatant.
8. Wash cells by resuspending in 200 μl PBS, centrifuging, and removing supernatant.
9. Repeat PBS wash.
10. Resuspend the cell pellet gently with 200 µl 1 × DSP and incubate at room temperature for 30 min.
11. To quench the crosslinker, add 4.1 µl of 1 M Tris HCl, pH 7.5 (final concentration 20 mM) and mix gently by pipetting.
12. Store fixed cells at 4 °C until they can be processed.
13. To decrosslink add DTT at 50 mM final concentration.

A video of the fixative preparation and DSP fixation process, showing the steps necessary to avoid precipitation of the DSP compound, is available at https://youtu.be/L2aiw14IXU4 (August, 2017).

### Fluorescence staining

Cells were stained with one of three staining combinations in four different experiments as detailed below. Experiments 1, 2, and 3 were performed with K562 cells (ATCC® CCL243™). In Experiment 1, all cells were stained with DNA stains - Hoechst 33342 “ThermoFisher” (2 µM final concentration, 20 minutes at room temperature) and propidium iodide “ThermoFisher” (3.75 µM final concentration, 20 minutes at room temperature) - where Hoechst permeates all cells and propidium iodide enters dead cells only. In Experiments 2, and 3 - half the cells were stained with CellTracker Green CMFDA “ThermoFisher” (1 µM final concentration, 30 minutes at room temperature) and LIVE/DEAD® Fixable Red Dead Cell Stain “ThermoFisher” (per manufacturer protocol), while the other half were stained with CellTracker Orange CMRA “ThermoFisher” (1 µM final concentration, 30 minutes at room temperature) and LIVE/DEAD® Fixable Red Dead Cell Stain “ThermoFisher” (per manufacturer protocol). CellTracker Orange and CellTracker Green stain all cells, whereas Fixable Red Dead Cell enters only dead cells. Post-staining, cells were thoroughly washed with PBS to remove unincorporated stain and re-suspended in PBS or 1x DSP for fresh and fixed cells respectively.

### Cell imaging

Imaging of cell suspensions and single cells captured on C1 integrated fluidic circuits (IFCs) was carried out using the Operetta High Content Imaging System (PerkinElmer).

### Single-cell workflow

Fresh or fixed cells (ATCC® CCL243™) were captured on the C1 system (Fluidigm) and processed using the SMARTer chemistry (SMARTer® Ultra™ Low RNA Kit for Illumina® Sequencing, Takara Clontech), according to the Fluidigm protocol, “Using C1 to Generate Single-Cell cDNA Libraries for mRNA Sequencing”, PN 100-7168 Rev H1 (August, 2017). The protocol was modified for fixed cell runs to incorporate a reverse crosslinking step as described - lysis Mix A was prepared with 10.5 µl Clontech Dilution Buffer plus 1 µl 1 M DTT rather than 11.5 µl Clontech Dilution Buffer. A subset of cDNA samples was run on Agilent 2100 Bioanalyzer (High Sensitivity DNA Analysis Kit as per manufacturer’s protocol). cDNA samples were selected after analysing the cell images from the IFCs, and prepared for sequencing using the Nextera XT DNA Library Prep Kit (Illumina) with our own in-house primers^7^. Up to 96 libraries were sequenced per experiment on a single Illumina HiSeq. 2500 100 bp paired-end sequencing lane in Experiment 1, or Illumina HiSeq. 4000 75 bp paired-end sequencing lane in Experiments 2, and 3.

### Library preparation from bulk samples

Bulk samples of 4000 K562 cells (fresh or DSP-fixed) were prepared simultaneously with the C1 run, as positive controls in each experiment. Both fresh and fixed cells were first incubated at 37 °C for 30 min in 50 mM DTT (DL-Dithiothreitol; Sigma, D9779-5G). RNA was extracted using the RNeasy^®^ Plus Micro Kit (Qiagen) and eluted in 14 µl of RNAse-free water. A total of 1 µl aliquots of the extracted RNA were processed using portions of the same RT and PCR reagent master mixes described for the tube controls in the C1 protocol above, followed by Nextera XT Library Generation. Nextera XT libraries from bulks were pooled and sequenced together with the single-cell libraries.

### RNA sequencing, mapping, gene counts and QC

RNA-seq reads were trimmed for Nextera and Illumina adapter sequences using skewer-v0.1.125^8^. Trimmed reads were mapped to a modified reference genome comprising the human genome *Homo sapiens* GRCh37 (human_g1k_v37, available at http://software.broadinstitute.org/software/genomestrip/node_ReferenceMetadata.html, August 2017) and ERCC RNA Spike-In Mix sequences (ThermoFisher), using HISAT2 version-2.0.0-beta^9^ with default parameters. Duplicate reads were marked using MarkDuplicates.jar implemented in Picard tools v1.92 (http://broadinstitute.github.io/picard/, August 2017). BAM alignments were name sorted with Samtools version 1.1^10^. Alignment metrics were calculated using CollectRnaSeqMetrics from Picard tools for full BAM files and for BAM files with potential PCR duplicates marked. Reads mapping uniquely to genes annotated in ENSEMBL release 75^11^ were counted using featureCounts^12^ implemented in subread-v1.5^13^. The distribution of reads among several categories – assigned reads (mapped uniquely to exons), multiple mapping, ambiguous mapping, no feature (mapped uniquely to intronic and intergenic regions) – was obtained from featureCounts summary. All further metrics were calculated using R core tools, version 3.2.4^14^. Read counts were normalized to counts per million (CPM) and the numbers of detected genes per sample were calculated by counting genes with at least 1 CPM. Unsupervised clustering of single cells based on gene expression values was performed using the consensus-clustering algorithm implemented in Bioconductor package, SC3 version 1.1.4^15^.

## Results and Discussion

### Cellular morphology maintained in fixed cells

**Figure 1:**
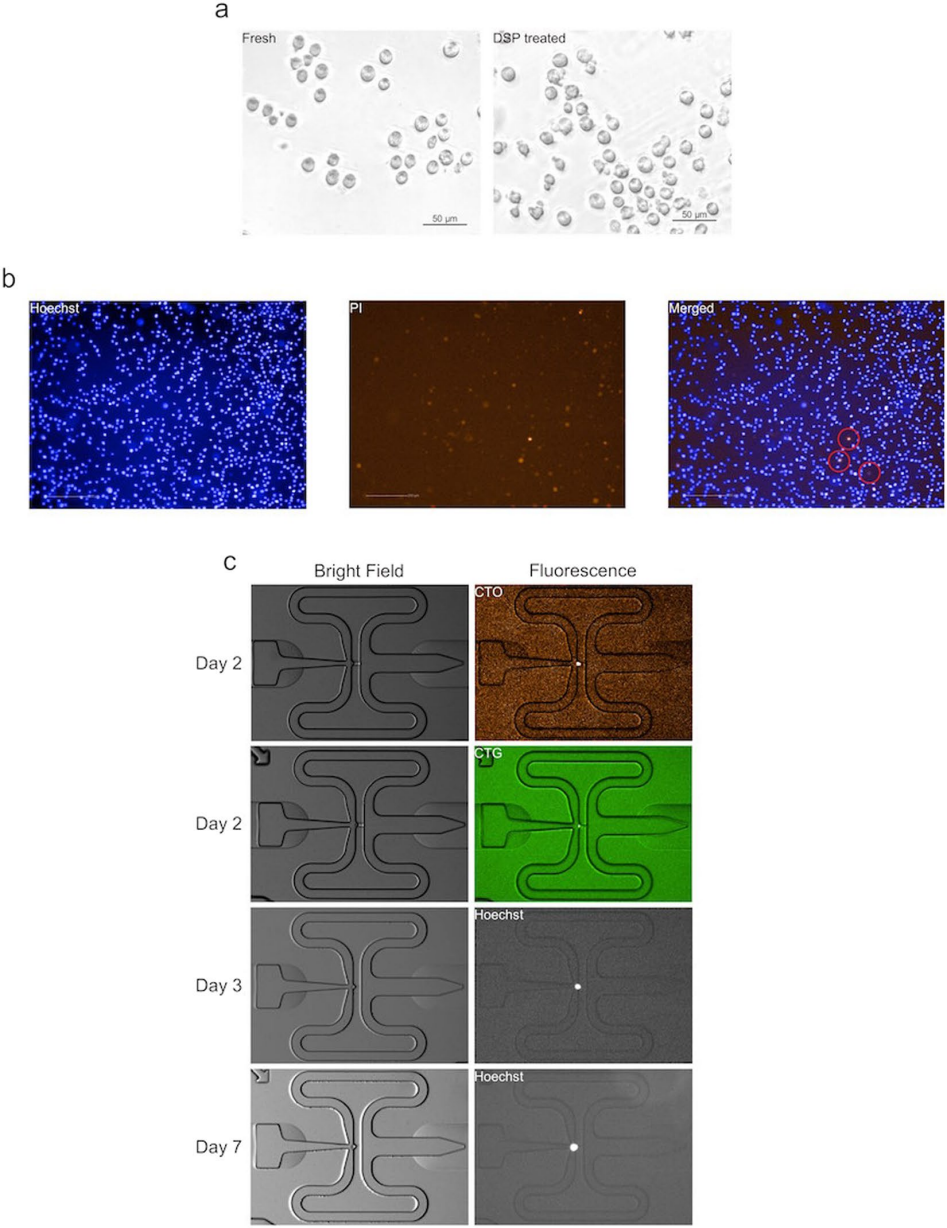
Imaging of fresh and DSP-fixed K562 cells. (**a**) Morphology of K562 cells before (left) and after (right) DSP treatment. DSP fixation did not cause detectable shrinkage or clumping. (**b**) Fluorescent staining of K562 cells after DSP treatment. In an overlay image of fixed K562 cells (right) stained with Hoechst 33342 (blue, left) and propidium iodide (PI; red, middle) before DSP treatment and then photographed 1 hour after fixation. Double-stained cells are circled in red (right). (**c**) Imaging of captured DSP-fixed K562 cells. Cells were captured on a Fluidigm C1 microfluidics IFC 2, 3 or 7 days after fixation, and imaged under bright field, and fluorescence for CTO (CellTracker Orange CMRA “ThermoFisher”), CTG (CellTracker Green CMFDA “ThermoFisher”) or Hoechst 33342.

The DSP fixation protocol was initially optimized using bulk cell samples by checking cell appearance and cDNA yields. Imaging of DSP-fixed cells showed similar morphology to fresh cells (Fig. 1a), with no obvious clumping or shrinkage upon fixative treatment. In case of cells stained using common dyes such as Hoechst 33342 or propidium iodide (PI), the cell-stain was retained in both fresh and fixed cells (Fig. 1b).

### Fixed single cells captured in C1 IFCs

K562 cells were captured on the C1 system in three independent experiments. In each experiment we compared fresh cells - captured immediately, without DSP treatment - with fixed cells of the same batch. Fixed cells were captured after 3 and 7 days (Experiment 1), or 2 days (Experiments 2 and 3) of fixative treatment. Cell isolation on the C1 IFC enables the collection of stain-related data by imaging fixed cells in the same manner as fresh captured cells, allowing resolution of nuclei in capture sites, and tracking of differentially stained sub-populations in experimentally mixed samples. Single fixed cells were readily captured in C1 IFCs with identification of differentially stained fixed cells similar to fresh cells (Fig. 1c).

### cDNA profiles from fixed single cells and fresh cells are comparable

**Figure 2:**
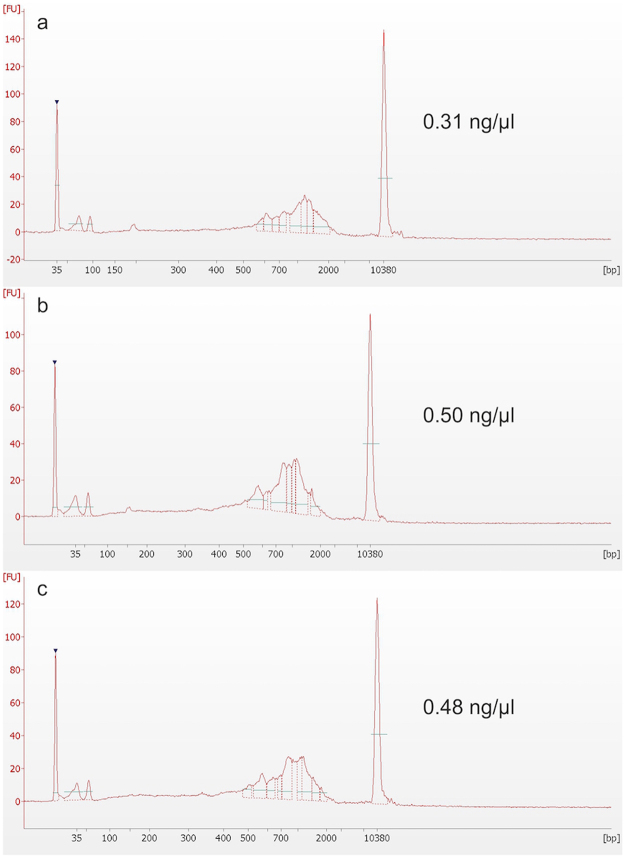
cDNA profiles of individual C1-captured K562 cells. Bioanalyzer (Agilent) profiles of cDNA from typical high-yielding cells failed to reveal an obvious effect of fixation. Example cells were isolated: (**a**) fresh, without fixation; (**b**) 3 days after DSP treatment; and (**c**) 7 days after DSP treatment. cDNA concentrations in ng/μl are shown.

**Figure 3:**
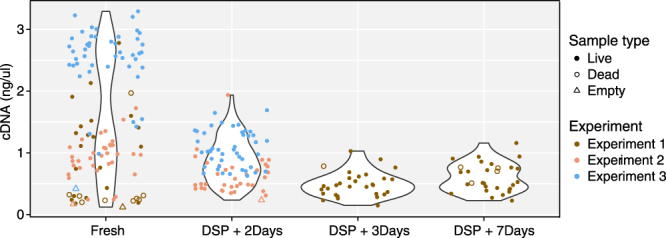
cDNA yields of individual C1-captured K562 cells. Cells were isolated fresh (without fixation; Experiments 1, 2, 3), and after DSP treatment for 2 days (Experiments 2 and 3), and 3, 7 days (Experiment 1). Cells that were PI+ at initial harvest (dead cells - hollow circle) were sequenced in Experiment 1 but excluded from sequencing in Experiments 2 and 3. Median yields from fixed cells were lower than yields from corresponding high-quality fresh cells. Drop in yields was not clearly related to time since fixation - Fixed cells kept for longer times (Day 7, Experiment 1) had lower yields than cells fixed for shorter period (Day 2, Experiment 3) but this also reflected differences in yield from fresh cells in the respective experiments.

The cDNA electropherogram profile did not differ significantly between single cells captured fresh and at different times after fixative treatment (Fig. 2). cDNA yields from fixed cells tended to be lower than those from fresh cells of the same experimental batch, but we observe higher variation in cDNA yield among fresh cells than among fixed cells (Fig. 3). In Experiment 1, all captured cells–PI+ (dead cells) and PI- (live cells)–were sequenced. In Experiments 2, 3 cells that stained positive for “Fixable Red Dead Cell Stain” (dead cells) were excluded from sequencing. As expected, non-viable (PI+) cells tended to have low yield, but intriguingly, there appeared to be very few such cells captured from fixed K562 samples. Moreover, the fixed PI+ cells had higher yields than fresh PI+ cells. This suggests a potentially important benefit of fixation, namely, limiting the handling of live cells by treatment with DSP before the capture step may better preserve the original biological state of analysed cells as compared to fresh cells. Cells from Experiment 3, both fresh and fixed, had higher cDNA yields than Experiments 1 and 2, indicating batch differences.

### Comparable gene expression profiles of fresh and fixed K562 bulks

**Figure 4:**
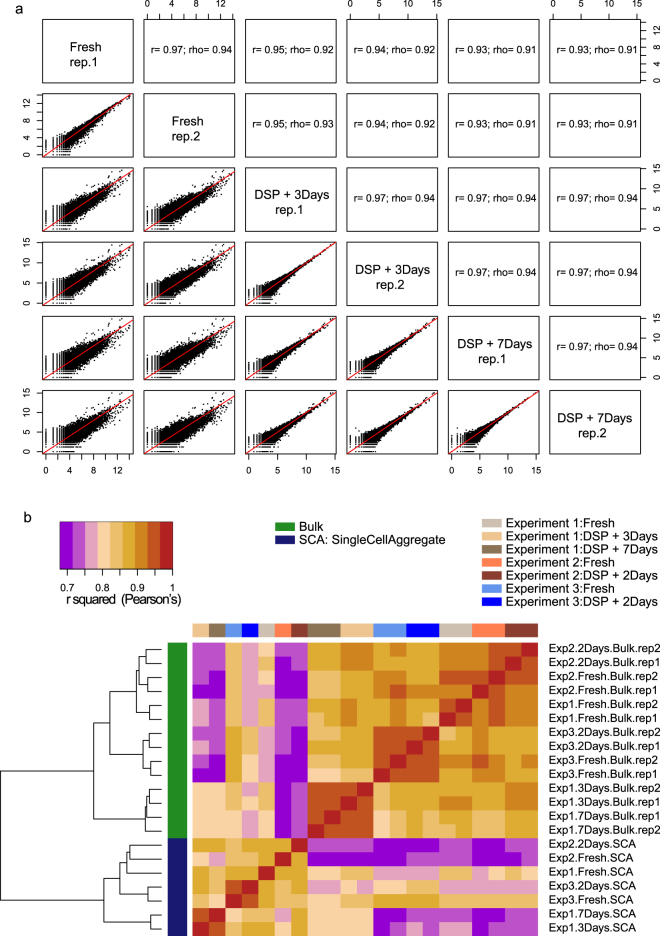
Correlation in bulk gene expression. (**a**) Scatterplots show pairwise correlation of gene expression values of Fresh and Fixed K562 bulks from Experiment 1 (above the diagonal: Pearson’s r and Spearman’s rank correlation coefficient; below the diagonal: scatterplots of log_2_ transformed CPM, counts per million, values). (**b**) Heatmap shows the correlation in gene-expression across bulk samples from all experiments and aggregate counts for single-cells. Gene expression profiles show high correlation between all bulks. The relative similarity of fixed cells stored for different times reveals signs of a systematic effect of fixation, while higher similarity within an experiment suggest that batch differences create more variation than fixative treatment.

We first evaluated the effect of fixative treatment on expression profile of RNA prepared from bulk cells. We see high pairwise correlation for overall gene expression (Pearson’s R > 0.93, p < 0.01) between fresh and fixed bulk samples (Fig. 4a, showing data from Experiment 1 only). The correlation among the fixed bulks from the same experiment, two biological replicates stored for 3 days and two biological replicates stored for 7 days after fixation, is comparable to that between fresh bulk biological replicates (R ~ 0.97, p < 0.01). We see similar trend of high correlation (*r*^2^ > 0.9) among all fresh and fixed K562 bulks across the three experiments (Fig. 4b). Likewise, biological replicates (pairs of fresh cell samples, or DSP fixed samples) show higher correlation with each other (*r*^2^ > 0.95). Expectedly, single cell aggregates (SCA- geometric mean of gene counts) cluster separately from bulks, emphasizing the variation captured in profiling from single-cells as opposed to mixed bulks. Highly correlated gene expression profiles across bulks indicate that cellular RNA preserved from the time of fixation can be harvested up to 7 days after fixation with minimal changes.

### Library complexity is maintained for fixed single cells

**Figure 5:**
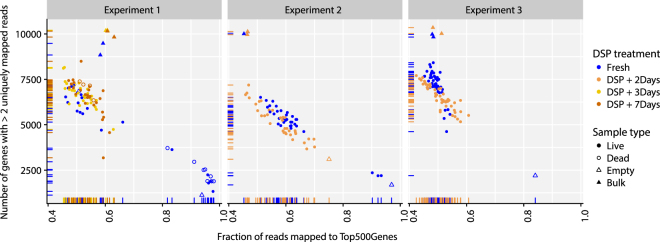
Transcript complexity: Counts of detected genes vs fraction of reads in top 500 genes. Measures of the recoverable complexity of transcripts show little difference between fresh and fixed cells, except that there were more PI+ fresh cells than fixed cells in Experiment 1. PI+ cells produced lower cDNA yields and failed standard complexity metrics.

Library complexity is a good indicator of the quality of RNA harvested in single-cell experiments. With approximately 5000–7000 genes detected and approximately 40–60% reads mapping to the top abundantly expressed 500 genes in the cell, both fresh and fixed K562 cells from Experiments 1–3 produced libraries of the similar complexity (Fig. 5). A subset of low-quality libraries with relatively few distinct transcripts and low cDNA yields (lower right of plots, Fig. 5), characteristic of PI+ dead cells and harvests that correspond to “empty” C1 IFC capture sites, was evident among fresh cells, but notably not among fixed cells. These data were removed from subsequent analyses (Table 1). The numbers of detected coding genes were not distinguishable between the remaining fresh and fixed cells (p > 0.1, Mann-Whitney U test).

**Table 1:** Summary of experiments: captured cells and bulk samples. Round brackets: samples removed at data QC.

	Experiment 1		Experiment 2		Experiment 3	
	Cells	Bulks	Cells	Bulks	Cells	Bulks
Fresh	29 (14)	2 (2)	34 (3)	2 (2)	46 (0)	2 (2)
DSP + 2Days	0	0	35 (2)	2 (2)	45 (0)	2 (2)
DSP + 3Days	30 (3)	2 (2)	—	—	—	—
DSP + 7Days	30 (4)	2 (2)	—	—	—	—

### Gene body coverage shows modest 3′ bias in fixed cells

**Figure 6:**
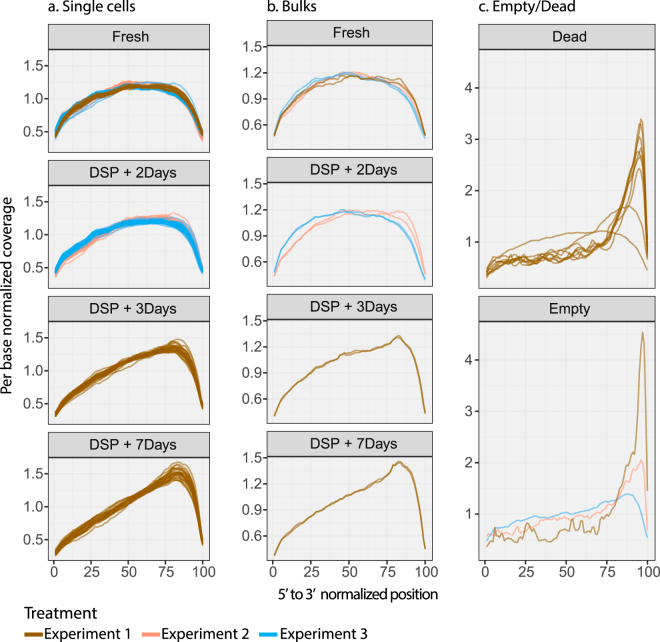
Bias in sequencing coverage across the gene body. Relative read-depth across all genes, normalised to the same length, reveals consistently greater bias in fixed cells than in fresh cells (**a**. Single cells, **b**. Bulks), which appeared to increase with time of storage after DSP treatment. Gene-body coverage profile for cells with low quality/complexity or empty C1 IFC capture sites show spiky coverage with very high 3′ bias (**c**). Only cells passing quality/complexity filters were included.

We used SMARTer chemistry, designed to retrieve full-length transcripts as cDNA, in this study. Plotting the relative coverage of reads along the (normalized) length of top expressed genes for each cell (Fig. 6a) reveals the expected lack of 5′-to-3′ bias in high-quality fresh cells. In fixed cells, however, there is a mild bias in coverage towards the 3′ ends of genes, which appears to increase with time of storage after DSP treatment. The bias is also present in fixed bulk samples (Fig. 6b). This bias is different in appearance from the irregular (‘spiky’), strongly 3′-biased plot characteristic of degraded RNA that we have observed in low-quality dead cells or empty C1 IFC capture sites (Fig. 6c). The absence of a reduction in gene-level complexity that accompanies the observed bias suggests that, while DSP treatment and storage may affect the retrieval of whole transcripts from cells, the priming efficiency of reverse transcription may be comparable to that in fresh cells and the method may be particularly suited to end-counting RNA-Seq methods.

### Experimental heterogeneity in cell-type profiles

**Figure 7:**
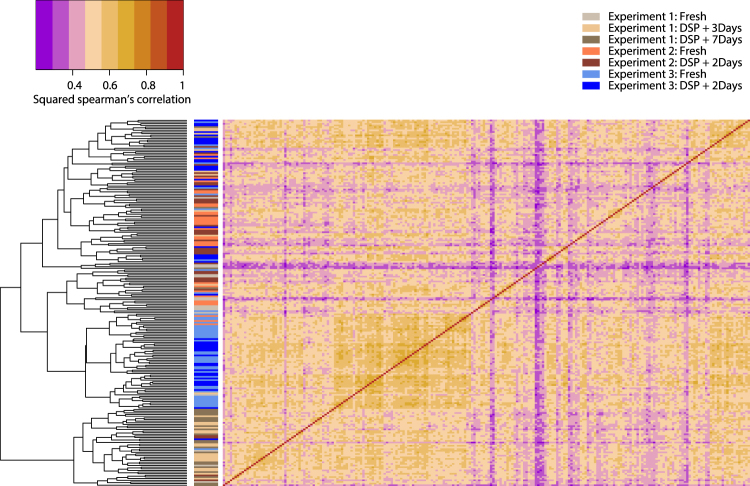
Pairwise expression correlation for all K562 cells. Heatmap of squared Spearman’s correlation coefficient of the expression of the top 500 genes observed across all experiments shows high similarity of expression profiles between fresh and fixed cells, especially for Experiment 3.

We compared single-cell expression profiles across several experiments and C1 runs to illustrate and contrast heterogeneity at several levels: within a sample of cells, between methods and storage time-points for the same sample, and between experiments. In comparisons between all K562 cells from three experiments (Fig. 7), the dominant factor distinguishing cells appears to be experimental batch (note: a distinct lot of K562s was used in Experiment 1 vs. Experiments 2 and 3), rather than whether the cells were fresh or fixed.

**Figure 8:**
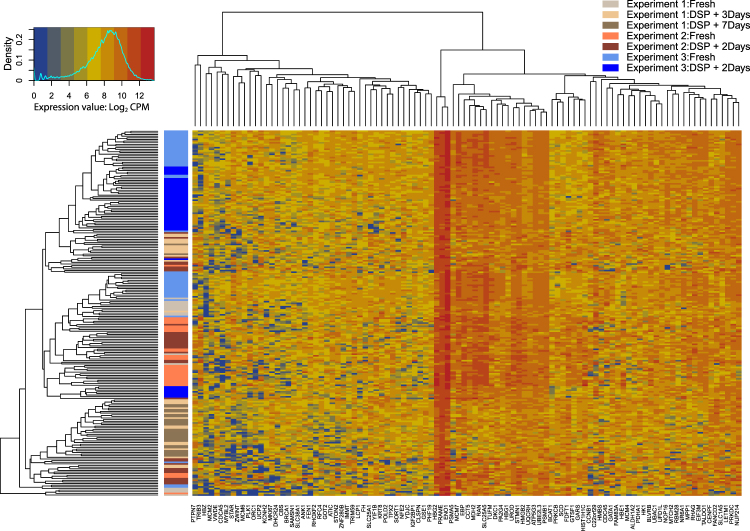
Unsupervised clustering comparison of 100 candidate genes with high expression in K562 cells. Along the y-axis, cells are arranged by highest similarity, revealing interspersed clustering between fresh and fixed cells and more distinct separation by experiment. The x-axis shows 100 marker genes for K562 cells.

The expression pattern of 100 candidate genes, used as K562 markers, also shows similar expression levels across all fresh and fixed cells with clustering by experiment batch over DSP treatment (Fig. 8). The observation in these clustering comparisons that fixed cells are often interspersed amongst fresh cells from the same experiment implies that the technical effects of DSP treatment on the observed transcriptome, at the gene level, may be of a smaller magnitude than batch effects in the data. This is good news for a researcher seeking to replace multiple experiments on different days with a single experiment in which fixed cells are processed in successive runs across several days.

## Conclusions

Single-cell genomics is leading to tremendous advancements in our understanding of the complexity of cellular models and cellular systems. Such studies may require the simultaneous production of many samples, whose single-cell analysis can be constrained by distance to and throughput of specialist facilities when cells need to be isolated and processed immediately to avoid sample degradation.

We have demonstrated that DSP treatment can be used for preserving cells for subsequent cell isolation and processing for single-cell RNA sequencing for at least several days after sample collection. Preserved cells are amenable to microfluidic manipulation and can be stained with most commonly used dyes for tracking and viability assessment. DSP cross-linking is easily reversed and compatible with downstream cell lysis and cDNA synthesis protocols. RNA-seq of fixed cells, captured and processed up to 7 days after DSP treatment, shows highly similar transcript complexity and transcriptome identity to fresh cells.

Cells preserved with DSP are stabilized at the time of sample collection and large numbers of fixed samples can be easily transported and subsequently processed for RNA-seq. This separation of the place and time of sampling from downstream processing enables complex study designs for single-cell experiments. DSP preservation has traditionally been applied to cell solutions as well as tissues, and DSP-treated samples are amenable for immuno-staining and FACS sorting^16,17^. Therefore, our method for cell preservation may also have utility for preservation of tissues at sampling before dissociation, and in combination with FACS sorting of stabilized cells.

### Data availability

The RNAseq data discussed in this publication have been deposited in NCBI’s Gene Expression Omnibus^18^. The sequencing data, experimental metadata and QC metrics are accessible through GEO Series accession number GSE98734 (https://www.ncbi.nlm.nih.gov/geo/query/acc.cgi?acc=GSE98734, August 2017).
